# Current and Emerging Roles of GLP1 Receptor Agonists Across the Spectrum of Left Ventricular Ejection Fraction in Heart Failure

**DOI:** 10.3390/biom15111574

**Published:** 2025-11-10

**Authors:** Simone Pasquale Crispino, Annunziata Nusca, Aurora Ferro, Riccardo Cricco, Martina Ciancio, Andrea Segreti, Ilaria Cavallari, Mario Sabatino, Luciano Potena, Gian Paolo Ussia, Francesco Grigioni

**Affiliations:** 1 Department of Medicine and Surgery, Università Campus Bio-Medico di Roma, 00128 Rome, Italy; simone.crispino@unicampus.it (S.P.C.);; 2 Department of Medicine and Surgery, Fondazione Policlinico Campus Bio-Medico, 00128 Rome, Italy; 3 Department of Biomedical Engineering, Intelligent Technology Laboratory for Health and Wellbeing, Università Campus Bio-Medico di Roma, 00128 Rome, Italy; 4 Department of Movement, Human and Health Sciences, University of Rome “Foro Italico”, 00135 Rome, Italy; 5 Heart Failure and Transplant Unit, IRCCS Azienda Ospedaliero-Universitaria di Bologna, 40138 Bologna, Italy

**Keywords:** glucagon-like peptide 1 receptor agonists, heart failure, heart failure with preserved ejection fraction, heart failure with reduced ejection fraction, cardio-kidney-metabolic syndrome, cardiovascular prevention, cardiometabolic disease

## Abstract

Glucagon-like peptide-1 receptor agonists (GLP1-RAs) have demonstrated significant cardiometabolic benefits, particularly in patients with type 2 diabetes and obesity. Their role in heart failure (HF) is gaining increasing attention, with growing evidence supporting their efficacy in HF with preserved ejection fraction (HFpEF). Recent trials have shown that semaglutide improves symptoms, functional capacity, and weight loss in patients with HFpEF. However, these trials did not demonstrate a reduction in HF hospitalizations or mortality. In contrast, tirzepatide has revealed a significant reduction in cardiovascular death and worsening HF events in patients with obesity-related HFpEF, suggesting broader cardioprotective effects. Concordantly, the benefit of GLP1-RAs in the setting of HF with reduced ejection fraction (HFrEF) remains uncertain. Although their mechanisms suggest potential advantages, particularly for patients with a cardiometabolic phenotype, clinical evidence supporting improvements in major clinical outcomes is lacking. Additionally, concerns regarding risk of increased HF hospitalizations, fluid retention and arrhythmic risk have led to a cautious approach in this population. As HF management continues to evolve, GLP1-RAs emerge as a promising yet complex therapeutic option. This review synthesizes the current evidence, highlights key knowledge gaps, and explores how these medications might be integrated into guideline-directed medical therapy (GDMT) to determine their optimal role across the LVEF spectrum in HF.

## 1. Introduction

### 1.1. Background

Heart failure (HF) represents a growing global health concern, with an estimated prevalence of over 64 million individuals worldwide. It is associated with significant morbidity, mortality, and healthcare expenditure, particularly in aging societies and those with high burdens of cardiovascular risk factors [1,2]. Despite advances in pharmacologic and device-based therapies, the overall prognosis of HF remains poor, with a 5-year mortality rate approaching 50% in many registries [3]. Historically, HF has been categorized according to left ventricular ejection fraction (LVEF). Current clinical practice stratifies patients into three major phenotypes: HF with reduced ejection fraction (HFrEF; LVEF < 40%), HF with mildly reduced ejection fraction (HFmrEF; LVEF 41–49%), and HF with preserved ejection fraction (HFpEF; LVEF ≥ 50%) [4]. Although LVEF-based classification is widely adopted, it tends to oversimplify the underlying pathophysiology, which is often multifactorial and heterogeneous.

HFrEF is typically the result of ischemic or non-ischemic myocardial injury, characterized by systolic dysfunction, ventricular remodeling, and neurohormonal activation. For this phenotype, several pharmacologic agents have demonstrated a mortality benefit and are standard components of guideline-directed medical therapy (GDMT), including beta-blockers, angiotensin receptor-neprilysin inhibitors (ARNIs), mineralocorticoid receptor antagonists (MRAs), and sodium-glucose co-transporter-2 inhibitors (SGLT2i) [4,5].

By contrast, HFpEF accounts for approximately half of all HF cases, yet therapeutic options remain limited. Currently, only SGLT2is have shown significant outcome benefits in large-scale trials [6,7].

HFpEF is closely associated with a constellation of metabolic comorbidities, including type 2 diabetes (T2DM), obesity, and chronic kidney disease (CKD) [8]. These patients frequently exhibit systemic inflammation, endothelial dysfunction, and microvascular impairment, contributing to impaired myocardial compliance and diastolic dysfunction.

### 1.2. Cardio-Kidney-Metabolic Syndrome

**Figure 1:**
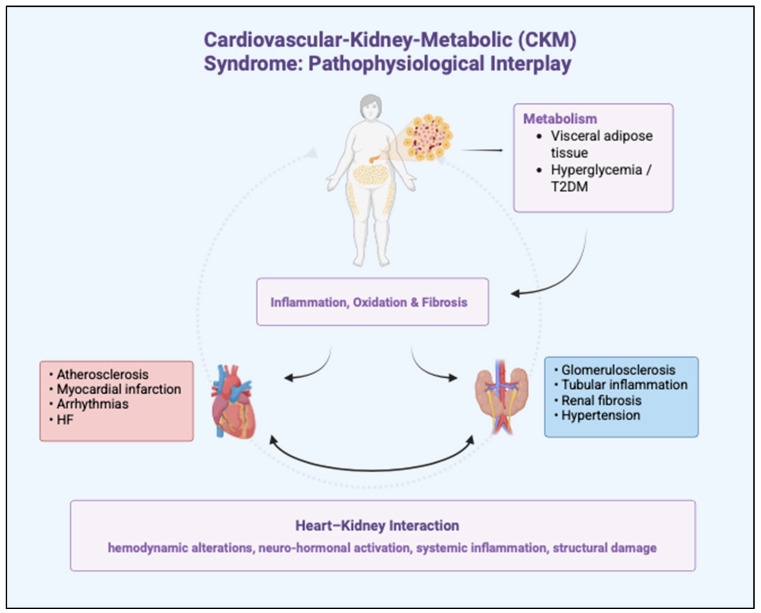
Schematic representation of the cardiovascular-kidney-metabolic (CKM) syndrome. The figure illustrates the central role of adipose tissue dysfunction and hyperglycemia/type 2 diabetes mellitus (T2DM) as upstream drivers of systemic inflammation, oxidation and fibrosis. These processes have been shown to exert detrimental effects on both the heart and kidneys, leading to a range of conditions. The bidirectional interaction between heart and kidneys is mediated through hemodynamic alterations, neuro-hormonal activation, systemic inflammation, and structural changes, thereby establishing a vicious cycle of reciprocal organ damage that characterizes the CKM syndrome.

Recently, the American Heart Association proposed the cardiovascular-kidney-metabolic (CKM) syndrome concept to capture the intersection of pathophysiological processes [9]. The CKM framework emphasizes multi-organ interactions and metabolic dysregulation in HF development, especially in HFpEF. Adipose tissue excess or dysfunction lies at the core of CKM syndrome. Visceral adipose tissue promotes inflammation and oxidative stress, affecting the arteries, heart, and kidneys [10]. Hyperglycemia, often progressing to type 2 diabetes mellitus (DMT2), is a risk factor for cardiovascular and renal damage, promoting inflammation and fibrosis. Inflammation drives atherosclerosis, myocardial infarction, glomerulosclerosis, and renal fibrosis. Ectopic epicardial fat deposition can cause arrhythmias, myocardial dysfunction, and coronary atherosclerosis, while kidney deposits contribute to hypertension. The heart-kidney interaction, mediated by hemodynamic alterations, atherosclerosis, neurohormonal activation, and inflammation, is crucial for CKM syndrome. Renal damage increases circulating volume, worsening HF progression, and vice versa [11]. With the rising burden of HFpEF and its metabolic comorbidities, novel interventions targeting systemic disease drivers are needed. The intersections of the cardiovascular-kidney-metabolic (CKM) syndrome are represented in Figure 1.

### 1.3. Cardiovascular Implications

Glucagon-like peptide-1 receptor agonists (GLP-1RAs) are a class of incretin-based therapies initially developed for glycemic control in patients with T2DM. These agents mimic the effects of endogenous GLP-1, a gut-derived hormone that stimulates insulin secretion, suppresses glucagon, delays gastric emptying, and promotes satiety. Pharmacological GLP-1RAs are resistant to enzymatic degradation by dipeptidyl peptidase-4 (DPP-4), thereby achieving sustained activity after subcutaneous or oral administration [12]. Beyond glucose regulation, GLP-1RAs exert multiple systemic effects with potential cardiovascular implications. Mechanistically, they improve insulin sensitivity, reduce body weight, lower systolic blood pressure, and ameliorate lipid profiles [13]. GLP-1 receptors are expressed not only in pancreatic islets but also in the central nervous system, gastrointestinal tract, kidneys, vascular endothelium, and myocardium, enabling diverse biological actions [14]. In the cardiovascular domain, the GLP-1 hormone can reduce ischemia–reperfusion injury through the activation of specific pathways such as PI3K/Akt, which reduces the Bax/Bcl-2 ratio in cardiomyocytes, cytochrome C release, and caspase activation, modulating apoptotic processes. Moreover, it enhances endothelial function by promoting nitric oxide (NO) production, resulting in vasodilatory, anti-inflammatory, and anti-atherosclerotic effects.

Thus, GLP-1RAs have been associated with consistent reductions in major adverse cardiovascular events (MACE) in patients with T2DM at high risk of atherosclerotic cardiovascular disease (ASCVD). Several studies reported significant reductions in cardiovascular death and non-fatal stroke, leading to their incorporation into international cardiometabolic guidelines [15,16,17]. More recently, the SELECT trial extended these findings to non-diabetic individuals with established CVD and overweight or obesity, confirming the cardioprotective effects of semaglutide independent of glycemic status [18].

Despite these early successes, the role of GLP-1RAs in the standalone HF management is less well defined In HFpEF, semaglutide and tirzepatide have demonstrated improvements in symptoms, functional capacity, and weight loss, particularly in patients with obesity-related phenotypes [19,20]. Conversely, trials in HFrEF have yielded inconclusive or even unfavorable results, with some data suggesting increased heart rate, arrhythmogenic potential, and fluid retention in vulnerable populations [21,22].

The clinical heterogeneity of HF, coupled with the pleiotropic mechanisms of GLP-1RAs, underscores the importance of a phenotype-driven approach to therapy. In this context, the integration of GLP-1RAs into HF care must consider individual patient profiles, comorbidities, and stage of disease progression.

### 1.4. Methods

This narrative review was designed to examine the therapeutic role of GLP-1RAs in HF, with an emphasis on phenotypic differentiation and cardiometabolic comorbidity.

Although GLP-1 receptor agonists have been extensively evaluated in patients with T2DM and atherosclerotic cardiovascular disease, their therapeutic potential in heart failure remains incompletely defined. Considering the peculiar pathophysiology of HFpEF and its typical association with metabolic diseases, existing literature has largely focused on their effects in HFpEF, particularly in the context of obesity-related phenotypes, while evidence in HFrEF and HFmrEF is limited and fragmented. Moreover, some uncertainty regarding safety in these categories remain to be clarified. Prior reviews have typically addressed GLP-1RAs as a homogeneous drug class without systematically distinguishing differences in pathophysiology, drug mechanisms, and outcomes across the LVEF spectrum. Given the growing recognition that heart failure represents a continuum rather than discrete phenotypes, a comprehensive descriptive and narrative synthesis that integrates clinical trial data, mechanistic insights, and cardiometabolic context across this spectrum is warranted.

The review adopts a phenotype-oriented framework, mirroring recent methodological approaches in translational cardiovascular research, and aligns with current strategies aimed at individualizing therapy based on LVEF and metabolic risk profiles. A structured literature search was conducted across PubMed, Scopus, and Web of Science for articles published up to June 2025. Search terms included combinations of “GLP-1 receptor agonists,” “heart failure,” “preserved ejection fraction,” “reduced ejection fraction,” “HFpEF,” “HFrEF,” “tirzepatide,” “semaglutide,” “liraglutide,” “cardiometabolic,” and “functional capacity.” Priority was given to randomized controlled trials (RCTs), meta-analyses, and mechanistic studies, particularly those reporting cardiovascular outcomes or HF-specific endpoints. Relevant guidelines and position papers were also reviewed to ensure alignment with current clinical recommendations. Evidence was synthesized according to HF phenotype—HFrEF, HFmrEF, and HFpEF—with particular attention to patient subgroups characterized by type 2 diabetes, obesity and chronic kidney disease. Mechanistic data were evaluated in parallel to support interpretation of clinical findings, especially in phenotypes where GLP-1RAs have shown divergent efficacy. No generative artificial intelligence tools were used for content generation. Minor linguistic refinements were performed using ChatGPT (GPT-5, OpenAI) under the full supervision and responsibility of the authors.

The primary aim of this review is to assess the role of GLP-1RAs across the entire HF spectrum, exploring both established and emerging indications. Secondary aims include outlining the underlying putative biochemical and pathophysiological rationale for GLP-1RA use in HF, examining potential interactions with other components of GDMT, and identifying unresolved clinical questions. This narrative review is intended to support a more tailored approach to pharmacologic therapy in HF, particularly within the context of cardiometabolic disease.

## 2. Focus on Mechanisms of Action of GLP1-RAs in HF

### 2.1. Systemic Metabolic Effects

GLP-1Rs are members of the B family of G protein-coupled receptors and are located in different human tissues, resulting in multiple systemic effects of GLP-1RAs [23,24].

GLP-1RAs have a hypoglycemic effect by stimulating insulin secretion in pancreatic β-cells, suppressing glucagon secretion in pancreatic α-cells and slowing gastric emptying [13]. They also promote feelings of satiety, and decrease appetite and stomach fullness, resulting in a lower calories intake [13]. Key aspects of this mechanism are related to GLP-1RAs and GLP-1R interaction in the hypothalamus arcuate nucleus and hindbrain [25]. Moreover, GLP-1RAs exhibit lipolytic activity when GLP-1R are present in high concentrations, such as in the visceral adipose tissue of morbidly obese patients with insulin resistance [26]. This was demonstrated in two randomized controlled trials that showed a decrease in epicardial adipose tissue in patients with T2D and obesity on liraglutide and exenatide, as assessed by echocardiography and cardiac MRI [27]. This is a relevant point, considering that epicardial and chest wall adiposity have been demonstrated to increase ventricular interdependence, thereby driving the development and progression of HFpEF [28]. Animal studies have also shown that GLP-1RAs therapy reduces reactive oxygen species production, macrophage and monocyte activation by oxidized LDL, adhesion molecules (VCAM-1, MCP-1, E-selectin, ICAM-1), foam cell formation, and endothelial apoptosis, reducing the atherosclerotic plaque’s necrotic core [29]. The GLP-1R stimulation in smooth muscle cells leads to a decrease in both their proliferation and migration into plaques [30]. Additionally, GLP-1R activation in endothelial cells results in the expression of more eNOS, providing greater nitric oxide and consequent vasodilation. GLP-1RAs also reduce systemic inflammation with decreases in CRP, *TNF-α*, *IL-6* and *IL-1β* levels, via effects on monocytes/macrophages [31].

Furthermore, GLP-1RAs are linked to improvement in lung function in chronic obstructive pulmonary disease (COPD) patients, as shown by improved spirometry results [32]. GLP-1RAs and SGLT2i reduce the risk of COPD exacerbations and respiratory infections, likely via anti-inflammatory and antioxidant pathways [32]. In experimental models, GLP-1RAs reverse lung fibrosis and enhance lung function [32].

### 2.2. Cardiovascular-Specific Effects

GLP-1RAs have been demonstrated to exert cardioprotective effects through a combination of metabolic, molecular, structural, and hemodynamic mechanisms [33]. At the myocardial level, they enhance glucose uptake and reduce oxidative stress, which improves cardiac energy efficiency, particularly during ischemic conditions or stress [34]. At the molecular level, GLP-1RAs activate pro-survival pathways such as PI3K/Akt and AMPK, which contribute to reduced cardiomyocyte apoptosis and improved cellular energy balance [35].

From a hemodynamic perspective, these agents have been linked to modest reductions in systemic blood pressure and decreased left ventricular wall stress; the effects can be partially attributed to reductions in both preload and afterload, potentially mediated by a combination of vasodilation and natriuresis [36]. Additionally, experimental evidence from animal models indicates that GLP-1RAs can attenuate left ventricular remodeling, reduce hypertrophy, and enhance diastolic function [37]. While some of these findings are derived from preclinical studies, they support a multifaceted role of GLP-1RAs in modulating cardiac function beyond glycemic control.

Beyond these preclinical findings, mechanistic insights may suggest that GLP-1RAs exert cardioprotective actions distinct from other components of GDMT [19,38,39]. SGLT2i primarily modulate preload and afterload through osmotic diuresis and improved ventricular loading [40]. In contrast, especially in the setting of HFpEF, GLP-1RAs target upstream metabolic and inflammatory pathways, improving myocardial substrate utilization and endothelial function [19]. This complementary profile provides a biological rationale for combining GLP-1RAs with standard HF therapies, particularly in cardiometabolic phenotypes.

### 2.3. Renal Protective Mechanisms

Although GLP-1 receptor expression in human kidneys is low in tubular cells, it is consistently observed in renal vasculature and juxtaglomerular cells, indicating that the renal effects of GLP-1RAs are likely to be indirect [41].

Clinical studies demonstrate that GLP-1RAs increase fractional sodium excretion, although this effect is attenuated in advanced kidney disease [42]. This natriuretic effect is hypothesized to be the result of the inhibition of sodium-hydrogen exchanger isoform 3 (NHE3) in the proximal tubule. This hypothesis has been demonstrated in preclinical studies, in which GLP-1RAs have been shown to reduce NHE3 activity via phosphorylation, leading to decreased sodium reabsorption [43].

However, human data suggest the presence of additional or alternative sites of action along the nephron. For instance, the lack of significant increase in lithium clearance, a marker of proximal tubular sodium handling, suggests that sodium loss may also occur in more distal segments of the nephron [42].

Evidence from cardiovascular and renal outcomes trials has demonstrated that GLP-1RAs reduce albuminuria and slow the progression CKD in patients with type 2 diabetes [44]. A recent meta-analysis demonstrated a 19% relative risk reduction in renal disease progression, and a dedicated renal outcomes trial confirmed benefit, primarily through reduced eGFR decline and cardiovascular risk [45,46].

Importantly, some renal benefits appear to be independent of glycemic, blood pressure, or weight changes, further suggesting the existence of additional mechanisms such as reduced inflammation, RAAS modulation, and improved endothelial and podocyte function [47].

## 3. Clinical Evidence for GLP1-RAs in HF Management

### 3.1. Stage A and B: Evidence in HF Prevention and Pre-HF

Stage A and B of the AHA classification refer to asymptomatic patients who are at risk for HF but without structural or functional heart disease (stage A) and patients with either structural heart disease, increased filling pressures in the heart or other risk factors with evidence of elevated natriuretic peptides or troponin (stage B) [5]. During the last years, HF management has gone through multiple changes, with a growing emphasis on prevention for patients with cardiovascular risk factors who are at risk of developing clinically relevant HF [48]. According to current guidelines, the management of risk factors is recommended to prevent the onset of clinically relevant HF (stage C). It consists of treating hypertension and diabetes and pursuing healthy lifestyle habits. For patients with diabetes and at stage A or B HF, the only recommended treatment by guidelines is the use of SGLT-2 inhibitors.

In this regard, multiple RCTs in patients with type 2 diabetes who are at risk for, or have established CVD, have shown that SGLT2i prevent HF hospitalizations compared with placebo [49,50,51,52].

Unlike HFrEF, which is primarily associated with ischemic injury or structural heart damage, HFpEF is strongly linked to metabolic disorders such as diabetes, obesity, and systemic inflammation, highlighting the importance of targeting these conditions for its prevention and management [53].

The LEADER trial analyzed the effects of a daily subcutaneous administration of 1.8 mg of liraglutide on CV outcomes and demonstrated a lower risk of cardiovascular events and deaths than placebo. Additionally, there was a noted reduction in HF hospitalization, although the difference was not statistically significant (HR 0.87 [95% CI 0.73–1.05]) [16]. The SUSTAIN-6 trial analyzed the effects of escalating doses of oral semaglutide in patients with type 2 diabetes and at high cardiovascular risk. The study demonstrated a reduction in cardiovascular death, nonfatal myocardial infarction and nonfatal stroke in patients treated with oral semaglutide but did not demonstrate a reduction in hospitalization for HF in the same group versus placebo [17]. A large meta-analysis of 54,092 patients analyzed data from seven randomized clinical trials involving patients with T2D treated with different GLP-1RAs. Of note, only 8460 patients (16%) had HF history. Compared with placebo, treatment with GLP1-RA did not reduce HF hospitalizations or cardiovascular death in patients with HF history. However, in those without a history of HF, there was a 16% relative reduction in hospitalizations and cardiovascular deaths. Furthermore, GLP1-RAs did not lead to decreases in all-cause death in patients with HF history, but they did demonstrate a mortality reduction among patients without HF history. These results suggest that GLP1-RAs may be beneficial in preventing new onset HF, but they do not seem to reduce hospitalizations and mortality in patients with T2D and established HF [54]. Despite the varying pathophysiological characteristics across the LVEF spectrum and the potential implications for heart failure treatment, only the EXSCEL study reported LVEF values, that was ≥40% in >90% of the patients with available echocardiographic data. This study investigated a weekly subcutaneous administration of 2 mg exenatide compared to placebo and did not show a significant reduction in MACE (HR 0.91; 95% CI 0.83–1.00; *p* = 0.06 for superiority) [55]. Interestingly, a sub-analysis of this trial demonstrated that the benefit of exenatide in reducing hospitalization for HF and mortality appears to be limited to patients without pre-existing HF [56].

The potential advantages of GLP-1RAs in HFpEF may also be linked to the benefits these drugs provide for all other chronic conditions associated with its development. Obesity, for instance, is an important risk factor for developing HFpEF, but it lacked specific pharmacological treatments for many years. GLP-1RAs demonstrated an important positive effect on obesity by decreasing the appetite and delaying gastric emptying, thereby leading to an improvement in energy balance [57]. In this regard, the SELECT trial was a large RCT conducted on 17,604 patients that examined the effects of semaglutide in patients without history of diabetes but with preexisting cardiovascular disease and affected by obesity and overweight (BMI ≥ 27 kg/m^2^). Patients received once-weekly subcutaneous semaglutide or placebo, starting with a dose of 0.24 mg, that was increased every 4 weeks (to once weekly doses of 0.5, 1.0, 1.7, and 2.4 mg) until the target dose of 2.4 mg was reached after 16 weeks. Semaglutide reduced the risk of MACE by 20% (HR 0.80 [95% CI 0.72–0.90]) and CVD death or HF hospitalization/urgent medical visit by 18% (HR 0.82 [95% CI 0.71–0.96]) [18].

Furthermore, CKD is often associated with metabolic disorders, especially diabetes, and commonly coexists with HFpEF. The FLOW trial investigated the effects of semaglutide in patients with DM and CKD. In this case, semaglutide was administered subcutaneously once weekly, starting at 0.25 mg for 4 weeks, increasing to 0.5 mg for another 4 weeks, and then maintained at 1.0 mg weekly, with dose adjustments allowed based on tolerability and participant-specific needs. Treatment with semaglutide was associated with a 24% reduction in the primary composite outcome including kidney failure, 50% or greater reduction in GFR, and kidney- or cardiovascular-related death. Moreover, there was a 29% reduction in CVD death and a 18% reduction in 3-point MACE (composed by CV death, non-fatal myocardial infarction and non-fatal stroke). The cumulative effect of these benefits was a reduction in all-cause death by 20% at 3 years [58].

Obesity and metabolic disorders are closely associated with systemic inflammation. Adipose tissue exerts an endocrine function through the secretion of a specific class of cytokines known as adipokines. Obesity induces adipocyte dysfunction, which elevates adipokine levels, consequently leading to left ventricular remodeling and heart failure with preserved ejection fraction (HFpEF) [59]. GLP-1RAs also demonstrated to reduce systemic inflammation, another cardiovascular protective mechanism [60].

Excessive activation of the renin–angiotensin–aldosterone system (RAAS) is well known to promote cardiovascular damage in HF by increasing blood pressure, vasoconstriction, cardiac hypertrophy, fibrosis, inflammation, vascular smooth muscle cell dedifferentiation, and reactive oxygen species production and reducing parasympathetic tone, nitric oxide production, and natriuresis [61]. There is controversial evidence about the usefulness of RAS modulation in HFpEF. However, some preclinical and clinical studies demonstrated that GLP-1RAs counteract angiotensin II, the primary bioactive peptide of the RAAS, and stimulate angiotensin-converting enzyme 2 (ACE-2), which transforms angiotensin II in angiotensin 1–7 that exerts vasodilatory and antifibrotic effect [62,63], even if this effect need clinical validation.

This evidence suggests that GLP-1RAs should be considered for HF prevention in at-risk patients with obesity, DM, and CKD. This is reflected by a Class IIb recommendation in the current U.S. guidelines for adults with T2DM and additional ASCVD risk factors [64].

**Figure 2:**
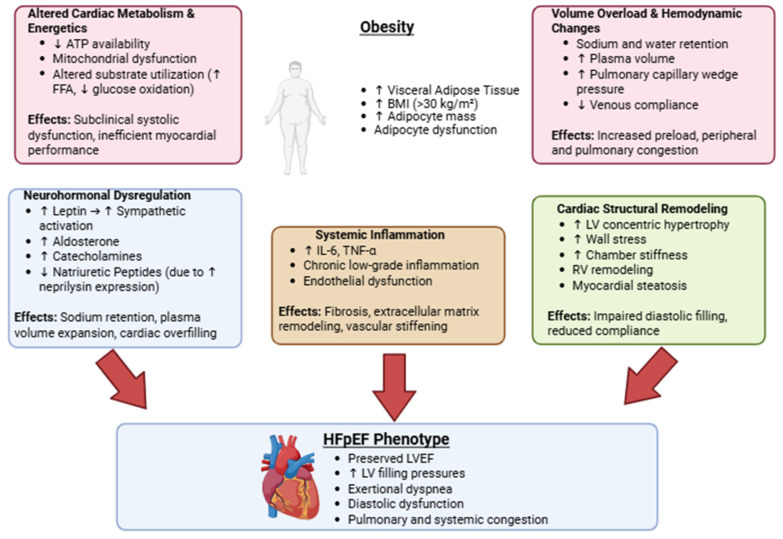
Main interactions between obesity and HFpEF: pathophysiological mechanisms. Obesity contributes to HFpEF through multiple interrelated processes, including altered cardiac metabolism, neurohormonal dysregulation, systemic inflammation, volume overload and hemodynamic changes, and structural cardiac remodeling. These pathways collectively lead to diastolic dysfunction, elevated filling pressures, and clinical manifestations of HFpEF.

Pathophysiological insights on HFpEF and obesity are resumed in Figure 2.

### 3.2. Stage C/D in HFpEF Management: Putative Implications

Stage C of the AHA classification refers to patients who have structural heart disease with current or previous symptoms of HF; in contrast, stage D pertains to patients whose HF symptoms significantly interfere with daily life, with recurrent hospitalizations despite GDMT optimization [5].

Stage C/D HFpEF has a more limited selection of evidence-based medications than HFrEF consisting of SGLT2 inhibitors (class IA), MRAs, RAS inhibitors, including angiotensin receptor blockers (ARBs) and angiotensin receptor-neprilysin inhibitors (ARNIs). However, these have only a class 2b recommendation in ACC/AHA HF guidelines because of their uncertain benefit, whereas no recommendation about this drugs has been released by current ESC guidelines [4,5].

However, recent trials have highlighted benefits that could derive from GLP-1RAs administration in patients affected by HFpEF in these stages.

The STEP-HFpEF trial was a randomized, double-blind, placebo-controlled trial that analysed the effects of weekly subcutaneous semaglutide in patients with obesity-related HFpEF. Inclusion criteria included left ventricular ejection fraction ≥45%, a body-mass index (BMI) ≥30 and a New York Heart Association functional class II, III, or IV. Patients with diabetes were excluded. Semaglutide treatment was initiated at a dose of 0.25 mg once weekly for the first 4 weeks, and the dose was escalated every 4 weeks to reach the maintenance dose of 2.4 mg by week 16. This trial demonstrated that patients treated with semaglutide experienced a reduction in HF symptoms, as measured by the KCCQ-CSS, and had a significant weight loss compared to placebo. Moreover, semaglutide therapy was associated with an increase in the 6 min walk distance and a reduction in CRP (C-reactive protein) levels. A reduction in NT-proBNP levels and systolic blood pressure was also reported in the treatment group, along with a lower number of adjudicated HF events (1 in the semaglutide group vs. 12 in the placebo group) [20]. These results are particularly relevant, as patients with HFpEF often suffer from a high burden of symptoms and physical limitations, and a poor quality of life. In a sub-study of the STEP-HFpEF trial, focused on an echocardiographic analysis, semaglutide appeared to improve LA (left atrium) volume, LV diastolic function, and right ventricular size compared with placebo. This evidence suggests that benefits of semaglutide on HFpEF symptoms may not only derive from weight loss but also from a disease modifying effect of this drug [65].

Similar results have been found in the more recent STEP-HFpEF DM trial, that analyzed the effects of subcutaneous semaglutide in a population with the same inclusion criteria but also presenting with T2D. The administration protocol of this study was identical to that of the STEP-HFpEF. Similarly, semaglutide led to a reduction in HF–related symptoms and physical limitations, a greater weight loss, and an increased 6 min walk distance (6MWT). Interestingly, weight loss was approximately 40% less than what was observed in the STEP-HFpEF trial, which supports the hypothesis that improvements in HF-related symptoms are not solely dependent by the magnitude of the weight loss. Similarly to the previous trial, there was a reduction in hospitalizations and urgent visits for HF, along with a reduction of NT-proBNP levels [20]. A successive pooled analysis of the two trials noted that patients with more severe HFpEF (as indicated by higher NT-proBNP concentration and the use of loop diuretic use) experienced larger improvements in KCCQ-CSS in the treatment group versus placebo than patients with less severe disease [66].

In November 2024, the SUMMIT trial was published, examining the use of tirzepatide, a long-acting agonist of glucose-dependent insulinotropic polypeptide and GLP1 receptors, in 731 patients with obesity and HFpEF. The two primary endpoints were a composite of adjudicated death from cardiovascular causes or a worsening heart-failure (WHF) event (assessed in a time-to-first-event analysis) and the change from baseline to 52 weeks in the KCCQ-CSS. Tirzepatide significantly reduced the composite of cardiovascular death or WHF events (36 patients, 9.9% vs. 56 patients, 15.3%; HR 0.62; 95% CI 0.41–0.95; *p* = 0.026) and improved clinical health status, with a 6.9-point greater increase in KCCQ-CSS at 52 weeks (*p* < 0.001). Among the secondary endpoints, this study demonstrated also a greater reduction in body weight (*p* < 0.001), an increase in the 6 min walk distance (*p* < 0.001) and a reduction in CRP levels (*p* < 0.001). Before this trial, no pharmacological therapy had definitively improved hard CV outcomes in HFpEF [19].

Subsequently, in a sub-analysis of the SUMMIT trial, tirzepatide was associated with reduced systolic blood pressure, estimated blood volume, urine albumin-creatinine ratio, N-terminal prohormone B-type natriuretic peptide levels, and troponin T levels. Additionally, tirzepatide increased the estimated glomerular filtration rate, suggesting a positive effect on circulatory volume expansion in patients with obesity-related HFpEF [67]. Furthermore, another CMR substudy of this trial has shown a reduction in LV mass, which correlated with the reduction in body weight and waist circumference, as well as a reduction in the paracardiac adipose tissue as compared with placebo. However, tirzepatide did not show benefit on LV end-systolic volume, LV function, LA volumes, function, or strain than placebo [38].

The more pronounced benefits observed with tirzepatide in the SUMMIT trial compared with those seen in the STEP-HFpEF program may be attributed to several factors. First, the study design differed substantially: SUMMIT trial incorporated hard cardiovascular outcomes, such as cardiovascular death and worsening HF events, whereas the STEP-HFpEF trials primarily focused on functional and symptomatic improvements. Second, differences in study populations may have contributed. Although the populations enrolled in both the STEP-HFpEF and SUMMIT trials were broadly similar, comprising patients with HFpEF and obesity, some subtle differences may have influenced the observed outcomes. In particular, SUMMIT trial included both diabetic and non-diabetic participants and patients with slightly higher baseline NT-proBNP levels and comorbidity burden, suggesting a population at greater cardiovascular risk. Beyond these design aspects, tirzepatide’s dual agonism of GIP and GLP-1 receptors may confer broader metabolic and anti-inflammatory effects compared to GLP-1 receptor activation alone, potentially accounting for the more robust results observed in SUMMIT.

Considering the IA recommendation on SGLT-2i use in patients with HFpEF, a recent retrospective cohort study of 7044 patients analyzed the possible benefit deriving from a combined therapy of GLP-1RAs and SGLT-2i. They found a lower risk of HF exacerbations, all-cause emergency department visits/hospitalizations, new-onset atrial arrhythmias, new-onset acute kidney injury, and pulmonary hypertension in GLP-1RAs plus SGLT2i cohort compared with the SGLT2i-only cohort, demonstrating a possible additive effect of a combination therapy [68].

GLP-1RAs could therefore represent an effective treatment to increase quality of life of patients with HFpEF and may also help to modify the natural history of HFpEF.

The following Table 1 resembles literature on GLP1ra in the setting of HFpEF.

### 3.3. HFrEF-Specific Mechanistic Rationale vs. Clinical Outcome Evidence

GLP-1RAs have several effects that could potentially be beneficial in HFrEF, including increased urinary sodium excretion, vasodilation, increased levels of endogenous natriuretic peptides and suppression of the renin-angiotensin system [69]. However, the prognostic significance of GLP-1RAs in patients with HFrEF remains to be elucidated, given the different pathophysiological substrates that characterize these two HF subtypes [70]. Main evidences on HFrEF are resumed in Table 2.

In this case, both mechanistic evidence and observations from randomized trials imply no discernible benefit on HF-related outcomes, and there is even some uncertainty regarding safety [21,71]. A meta-analysis of the FIGHT and EXSCEL trials indicated that GLP-1RAs are linked with an increased risk of HF hospitalization in patients with HF and an LVEF < 40% [72]. The LIVE study also supports an higher risk of adverse events with GLP-1RAs in HFrEF; specifically, there was a significant increase in the risk of serious cardiac events with liraglutide, including one death and one case of worsening of HF over 24 weeks [22]. Furthermore, albiglutide was administered to patients with T2DM and HF. No significant differences were observed in LVEF, brain natriuretic peptide, the 6MWT, myocardial glucose, or oxygen use at 13 weeks when comparing 30 patients receiving placebo to 27 patients receiving albiglutide 30 mg. However, the trial was too small and short to adequately evaluate outcomes [73]. Nevertheless, it is imperative to interpret these results as exploratory and hypothesis-generating, as they are based on a subgroup of the EXSCEL trial with available LVEF data and on small trials with limited follow-up periods. Concordantly, data from the Swedish HF Registry, linked with the National Diabetes Registry and other national registries, showed that the use of GLP-1RAs was more effective in more severe T2DM, reduced EF and obesity. However, GLP-1RAs therapy was not associated with a higher risk of HF hospitalization and cardiovascular death but with longer survival and fewer major cardiovascular adverse events [74]. A retrospective analysis of the Clalit Health Services Heart Failure Outcomes Cohort also revealed that diabetic patients with HFrEF exhibited a relatively strong response to treatment with GLP-1RAs, which was associated with improved clinical outcomes [75]. Therefore, there is a clear need for dedicated randomized controlled trials to evaluate the effects of GLP-1RAs in patients with HFrEF [76].

Stage D defines the advanced stage of the HF disease, when patients experience persistent and severe symptoms that remain unresponsive to optimal medical therapy [5]. This phase is characterized by significant morbidity and mortality. To date, no clinical trials have assessed the large-scale use of GLP-1RAs in this patient population. It has been established that a proportion of patients suffering from advanced HF also experience cachexia [77]. Consequently, further weight loss induced by GLP-1RAs may not be beneficial for these individuals [78].

**Table 2:** Evidence in HFrEF on hard CV endpoints in trials. N/A: non applicable.

Trial	Intervention	MACE Reduction	CV Death Reduction	HF Hospitalization Reduction
LEADER (2016) [16]	Liraglutide	Yes	Trend	Not statistically significant
SUSTAIN-6 (2016) [17]	Semaglutide	Yes	Yes	No
SELECT (2023) [18]	Semaglutide	Yes (20%)	Yes	Yes
FLOW (2024) [58]	Semaglutide	Yes (29%)	Yes	Yes
HARMONY (2018) [73]	Albiglutide	N/A	N/A	Yes

### 3.4. Concerns Regarding Fluid Retention and Arrhythmic Risk

One important concern regarding the use of GLP-1RAs in patients with HFrEF is the risk of fluid retention, as highlighted in a study involving liraglutide. In this randomized crossover trial, patients with an eGFR below 30 mL/min/1.73 m^2^ exhibited a significantly diminished sodium excretion when compared to patients with an eGFR above 60 mL/min/1.73 m^2^. The blunted natriuretic response in patients with advanced kidney disease was associated with transient increases in blood pressure and signs of fluid accumulation following liraglutide administration [79]. These findings are not unexpected, since GLP-1 has been shown to increase natriuresis by inhibiting many ion exchangers, which decrease as chronic kidney disease (CKD) progresses and interstitial fibrosis and tubular atrophy occur. Therefore, liraglutide’s natriuretic effect was minimal in patients with eGFR < 30 mL/min/1.73 m^2^, but a significant decrease was reported in the thoracic fluid index [80]. Nevertheless, the need for caution appears to be driven more by concomitant CKD than by HFrEF itself.

Another concern is the potential for arrhythmias in HFrEF. In the LIVE trial, liraglutide increased heart rate by ~6 bpm and was associated with a higher incidence of serious cardiac side effects, including ventricular tachycardia and atrial fibrillation requiring cardioversion, compared with placebo [22]. Similarly, the FIGHT study reported a trend toward more arrhythmic and HF-related events, raising concern about worsening outcomes in susceptible HFrEF patients [72].

A potential underlying mechanism involves the intracellular activation of adenylate cyclase by GLP-1RAs, leading to increased cAMP levels and subsequent activation of protein kinase A (PKA) [81]. This signaling cascade has been demonstrated to enhance L-type calcium channel activity and promote calcium entry into the cell [82]. In patients with HFrEF, this pathway may present challenges, given that their myocardium is already susceptible to calcium handling abnormalities, including sarcoplasmic reticulum Ca^2+^ leak and dysfunctional ryanodine receptors (RyR2) [83,84]. Moreover, the persistent activation of the sympathetic nervous system, which is typical in HFrEF, further amplifies cAMP-PKA signaling, thereby intensifying the intracellular calcium overload [84]. Excess calcium has been demonstrated to induce early and delayed afterdepolarizations, thus increasing the susceptibility to arrhythmias [82].

Nevertheless, further research may be required as GLP-1RAs-induced weight loss may also help reduce the risk of arrhythmias, since obesity and obesity-related conditions increase the likelihood of developing arrhythmias and sudden cardiac death [85].

### 3.5. Potential Benefits in Patients with Overlapping Cardiometabolic Conditions in HFrEF

GLP-1RAs were originally developed for the treatment of diabetes. They have been very effective in the management of obesity, reducing weight by 10–20%. These agents have also been found to reduce the risk of atherosclerotic cardiovascular disease outcomes, both in individuals with obesity and in those with diabetes [54]. Obesity significantly increases the risk of HF, often through complex physiological mechanisms, which can exceed the risk associated with atherosclerotic cardiovascular disease [21]. Obesity has been shown to occur in HFrEF cases, but the extent to which it contributes to HF and patient outcomes is debated. The role of obesity is hypothesized to be less significant than other factors, such as neurohormonal stress, hemodynamics, congestion, skeletal muscle dysfunction and sarcopenia [86,87]. Thus, dedicated trials are needed also in HFrEF [86].

## 4. Considerations for Clinical Practice

### 4.1. Current Indications and Patient Selection

Currently, this class of medications should be considered for patients with T2D who are at high cardiovascular risk, as well as for obese patients with a BMI ≥ 30 kg/m^2^, or for overweight patients with a BMI ≥ 27 kg/m^2^ and comorbidities such as hypertension, dyslipidemia, etc. In these patients, weight loss and glycemic control are considered beneficial in preventing the progression of HF, as reported by the latest ESC and ACC/AHA guidelines. The use of these agents in patients with established HF with reduced or preserved ejection fraction is more debated [88].

The latest 2024 consensus published by the ESC emphasizes the importance of managing metabolic dysregulation in the prevention of adverse cardiovascular events. It underscores the need to treat obesity as a chronic disease requiring intervention through primary and secondary prevention strategies that include the use of GLP-1RAs. Specifically, their use is currently recommended as indicated by the class of recommendation: (1) for patients with T2D, obese or overweight, with the goal of weight loss (Class IIa, Level of Evidence (LoE) B); (2) for patients with T2D and atherosclerotic disease, aiming to reduce cardiovascular risk irrespective of baseline or target glycated hemoglobin levels (Class I, LoE A); (3) for overweight (BMI ≥ 27 kg/m^2^) or obese non-diabetic patients with chronic coronary syndrome, with the goal of reducing the risk of cardiovascular mortality, acute myocardial infarction, or stroke (Class IIa, LoE B) [89].

The pleiotropic cardiometabolic effects of GLP-1RAs suggest their potential to complement established GDMT agents. In cases of HFpEF associated with obesity or T2DM, the early combination of GLP-1RAs with SGLT2i may result in additive benefits regarding metabolism, renal function and decongestion. These benefits are achieved through putative distinct mechanisms: GLP-1RAs primarily improving systemic inflammation and adiposity, and SGLT2is promoting natriuresis and improved ventricular loading. Conversely, in the context of HFrEF, where ARNI therapy constitutes the fundamental element of GDMT, the utilization of GLP-1RA should be contemplated primarily in stable patients with obesity or diabetes, consequent to hemodynamic optimization, in order to prevent the occurrence of overlapping hypotensive or volume effects. However, it is evident that the optimal sequencing and combined use of these agents across HF phenotypes must be further investigated through future randomized trials.

**Figure 3:**
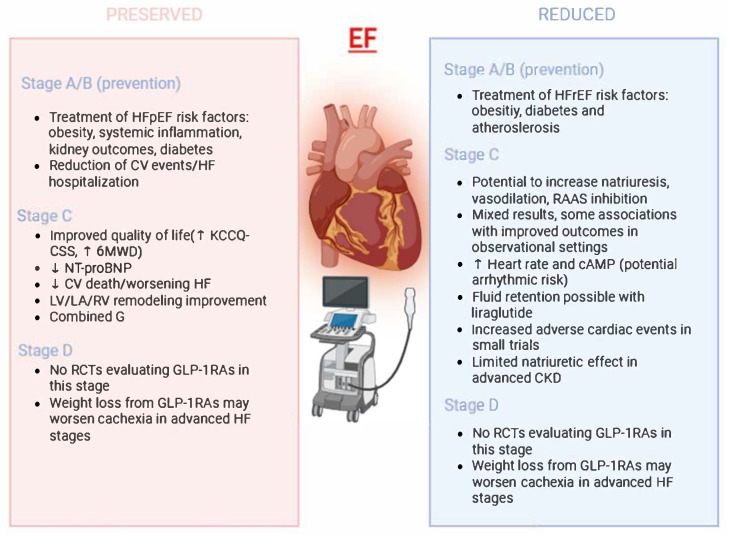
Central illustration. The putative effect along the journey of HF among the spectrum of LVEF: stage-specific recommendations for GLP1-RA use in HF and its prevention. In HFpEF, GLP-1RAs have been associated with improvements in quality of life, functional capacity, biomarkers, and cardiac remodeling. In contrast, evidence in HFrEF is less consistent, with reports of possible adverse hemodynamic and arrhythmic effects. In both phenotypes, no randomized controlled trials are available in advanced disease stages, where GLP-1RA-induced weight loss may exacerbate cachexia. Abbreviations: 6MWD: Six-Minute Walk Distance; cAMP: Cyclic Adenosine Monophosphate; CKD: Chronic Kidney Disease; CV: Cardiovascular; GLP-1RAs: Glucagon-Like Peptide-1 Receptor Agonists; HF: Heart Failure; HFpEF: Heart Failure with preserved Ejection Fraction; HFrEF: Heart Failure with reduced Ejection Fraction; KCCQ-CSS: Kansas City Cardiomyopathy Questionnaire-Clinical Summary Score; LA: Left Atrium; LV: Left Ventricle; NT-proBNP: N-terminal pro-B-type Natriuretic Peptide; RAAS: Renin–Angiotensin–Aldosterone System; RCTs: Randomized Controlled Trials; RV: Right Ventricle.

Figure 3 (Central Illustration) presents the highlights of GLP1ra along the journey of HF.

### 4.2. Follow-Up, Side Effect Management, and Risk Stratification

The clinical use of GLP-1RAs requires careful monitoring, proactive management of adverse effects, and personalized risk stratification to ensure therapeutic efficacy and safety.

During the initial phase of treatment, patient education plays a crucial role in promoting appropriate medication use and adherence to dietary recommendations, both of which are essential to minimize adverse effects and enhance treatment compliance. Therapy is typically initiated at the lowest available dose, which is then gradually titrated upward over the course of several weeks based on individual tolerability. In this phase, regular monitoring of renal and hepatic function, as well as glycemic levels (considering the low risk of hypoglycemia), is essential to assess the response to the drug and identify any adverse reactions early [90].

The most common side effects of GLP-1RAs are gastrointestinal in nature, such as nausea, vomiting, diarrhea, and constipation. These manifest especially during the first weeks of therapy and can be mitigated through appropriate dietary advice. Over time, they tend to reduce due to tachyphylaxis on the vagus nerve [91].

Rarer but potentially serious events, such as acute pancreatitis, acute cholecystitis, and the risk of medullary thyroid carcinoma, necessitate careful patient selection, avoiding use in individuals with a personal or family history of thyroid neoplasms or neuroendocrine tumors, as well as pancreatitis. Furthermore, the use of GLP-1 RAsis not recommended in pregnant women [92,93].

Emerging observational data have shown a potential elevated risk of neovascular age-related macular degeneration (nAMD) in patients with type 2 diabetes. One population-based cohort study [94] found that among patients aged ≥ 66 years, the incidence of nAMD was 0.2% in GLP-1 RA users versus 0.1% in matched non-users (adjusted hazard ratio ~2.2), although the mechanism remains unclear. Hypotheses include rapid glycaemic reduction, altered retinal perfusion, or direct retinal GLP-1 receptor effects. Given the relatively low absolute incidence but significant relative increase, clinicians should consider ophthalmic monitoring in higher-risk individuals with T2DM when prescribing GLP-1RAs.

In addition, possible adverse events are mainly related to the injection site. Other less common side effects include headache, tremors, and mild tachycardia.

Finally, particular caution is warranted when prescribing GLP-1 receptor agonists to patients over the age of 75, to prevent the potential development or worsening of sarcopenia [95].

In any case, risk stratification should also consider cardiovascular and renal comorbidities, given the favorable profile of some GLP-1RAs in these contexts. The integration of these factors into a multidisciplinary approach, based on updated guidelines and adapted to the individual patient’s characteristics, represents a fundamental prerequisite for optimizing the benefits of GLP-1 therapy [96].

## 5. Ongoing and Upcoming Clinical Trials

### Studies Focused on HFpEF, Particularly in Obesity-Driven and Metabolic HF Phenotypes: Combination Therapies and Dual-Agonist Agents

In recent years, dual- and multi-agonist therapies have emerged as a promising strategy to address the metabolic and inflammatory components of HF, particularly in patients with obesity or type 2 diabetes. These strategies simultaneously engage incretin pathways to promote weight loss, improved glycemic control, and cardiovascular benefits, with growing interest in their use for HFpEF [97].

Tirzepatide, a dual agonist of GLP-1 and GIP receptors, is the most advanced compound in its class: in the SUMMIT trial, among patients with HFpEF and obesity or type 2 diabetes, it lowered the risk of HF-related adverse events and cardiovascular death [19]; in the SURMOUNT-5 trial, it outperformed semaglutide in reducing body weight and waist circumference at week 72 in participants with obesity but without diabetes, reinforcing its cardiometabolic potential [98].

Cangrisema, a fixed dose combination of semaglutide and Cagrilintide (a long-acting human amylin analogue with affinity for three amylin receptors AMY1R, AMY2R, and AMY3R and the calcitonin receptor), acts centrally to modulate appetite regulation and, in REDEFINE 1 and 2 trials improved metabolic profiles in obese and diabetic patients. The ongoing REDEFINE 3 trial will further assess its impact on MACE in patients with established cardiovascular disease [99,100].

Similarly, the GLORY-1 trial investigated the dual GLP-1/glucagon agonist mazdutide in adults with overweight or obesity, demonstrating significant weight loss and metabolic improvements [101].

In this context, efinopegdutide, a dual GLP-1/glucagon agonist, has been studied in non-alcoholic fatty liver disease (NAFLD), a condition frequently associated with HFpEF, in a phase IIa trial showing greater antagonist reduction in liver fat content when compared with semaglutide (NCT04944992).

MariTide (also known as maridebart cafraglutide) is another dual agonist that utilizes a distinct mechanism compared to the conventional GLP-1/GIP. It employs a combination of a GLP-1 agonist and a monoclonal antibody antagonist of the GIPR receptor, with a monthly administration schedule. The MARITIME HF trial, in patients with HFpEF or HFmrEF and obesity, will investigate mortality, hospitalization, NT-proBNP levels, cardiac function, and quality of life (NCT07037459).

Ongoing phase 3 studies of survodutide (BI 456906), a dual GLP-1/glucagon receptor agonist, primarily assess its metabolic and weight-reducing effects. The SYNCHRONIZE-CVOT trial will specifically investigate cardiovascular safety and efficacy in patients with overweight or obesity and established cardiovascular disease or related risk factors [102].

Taken altogether, these findings underscore the growing potential of dual/multi-agonist therapies to target the cardiometabolic phenotype closely linked to HFpEF; importantly, because most trials have predominantly enrolled patients with obesity, additional mechanisms underlying the observed benefits remain to be clarified. For example, despite the expected rise in NT-proBNP with weight loss, pivotal studies show semaglutide produces a significantly greater reduction in NT-proBNP and fewer heart-failure events, suggesting decongestive and favorable hemodynamic effects that may be particularly consequential beyond weight loss.

## 6. Conclusions

GLP-1RAs have demonstrated substantial cardiometabolic benefits beyond glycaemic control, including weight reduction, anti-inflammatory effects, improved endothelial function, and favourable modulation of adipose tissue distribution. In patients with T2DM and high cardiovascular risk, large-scale randomized controlled trials have consistently shown that GLP-1RAs reduce MACE, particularly non-fatal stroke and cardiovascular mortality. In the context of HF, however, the clinical utility of GLP-1RAs appears to be highly phenotype-dependent. The most robust evidence supports their use in HFpEF, particularly among individuals with coexisting metabolic syndrome or T2DM, who constitute most of this population. Trials have shown significant improvements in functional status, quality of life, body weight, and exercise tolerance. While reductions in HF hospitalizations and cardiovascular death have not been consistently demonstrated in HFpEF cohorts, the data suggest a potential for symptom modification and disease stabilization in selected patients. Ultimately, tirzepatide has extended this therapeutic promise, showing significant reductions in HF related outcomes even in non-diabetic individuals with obesity, thereby confirming the class potential for broad cardiovascular risk reduction.

Conversely, the available evidence for HFrEF remains limited, inconsistent, and potentially unfavourable, although these findings arise predominantly from secondary analyses of trials not specifically designed for HFrEF.

From a preventive standpoint, GLP-1RAs appear to exert the greatest benefit in early stages of HF, particularly in stage A, where individuals are asymptomatic but at increased risk due to obesity, insulin resistance, or other cardiometabolic factors. In this setting, GLP-1RAs may attenuate or delay the development of HFpEF, supporting their role as upstream disease-modifying agents. However, for stages B, C, and D, evidence remains scarce or indirect, and no trial to date has demonstrated clear benefits on HF progression or outcomes across these stages, especially in HFrEF, where GLP-1RAs have not shown preventive efficacy.

In summary, from a therapeutic point of view, GLP-1RAs should be regarded as supplementary agents within the wider framework of HF treatment. GLP-1RAs should not be considered first-line therapy for overt HF, as they represent a valuable tool in the prevention and early modulation of HFpEF in patients with metabolic risk. Their favourable safety profile and systemic effects make them well-suited for integration into stage-specific, phenotype-driven management strategies, particularly when combined with other cardiometabolic agents such as SGLT2 inhibitors. Future studies are warranted to delineate their role across the HF continuum, define optimal timing of initiation, and clarify their potential interactions with established guideline-directed medical therapies.

## Figures and Tables

**Table 1:** Resumes current evidence on the use of GLP1ra in HFpEF. General evidence in HFpEF.

Study/Trial	Population	Intervention	Main Findings	Key Outcomes
LEADER (2016) [16]	T2DM + high CV risk	Liraglutide	Reduced CV events and mortality; no statistically significant reduction in HF hospitalizations	HR for MACE: 0.87 (95% CI 0.78–0.97, *p* < 0.001) HR for CV deaths: 0.78 (95% CI 0.66–0.93, *p* = 0.007)
SUSTAIN-6 (2016) [17]	T2DM + high CV risk	Semaglutide	Reduced MACE; no benefit on HF hospitalization	HR for MACE: 0.74 (95% CI 0.58–0.95, *p* < 0.001)
EXSCEL (2019) [55]	T2DM, ≥90% with LVEF ≥ 40%	Exenatide	No benefit in HF subgroup; benefit in those without HF	HR for first occurrence of any component of the composite outcome of death from MACE: 0.91 (95% CI 0.83–1.00, *p* < 0.001 for non-inferiority and *p* = 0.06 for superiority)
Ferreira et al. Meta-analysis (2023) [54]	54,092 T2DM patients, 16% with HF	Mixed GLP-1RA	Benefit in patients without HF; no benefit in established HF	GLP1-RA did not reduce the composite of HF hospitalization or cardiovascular death in patients with HF history: HR 0.96 (95% CI: 0.84–1.08), but reduced this outcome in patients without HF history: HR 0.84 (95% CI: 0.76–0.92). GLP1-RA did not reduce all-cause death in patients with HF history: HR 0.98 (95% CI: 0.86–1.11), but reduced mortality in patients without HF history: HR 0.85 (95% CI: 0.79–0.92).
SELECT (2023) [18]	Obese patients, no diabetes	Semaglutide	↓ MACE by 20%; ↓ HF-related events by 18%	HR for MACE: 0.80 (CI 0.72–0.90, *p* < 0.001)
FLOW (2024) [58]	T2DM + CKD	Semaglutide	Reduced renal and CV outcomes; ↓ CVD death (29%)	HR for major kidney disease events: 0.76 (95% CI: 0.66–0.88) HR for MACE: 0.82 (95% CI 0.68–0.98, *p* = 0.029)
STEP-HFpEF (2023) [20]	Obese HFpEF (no T2DM), LVEF ≥ 45%	Semaglutide	Improved symptoms (KCCQ-CSS), 6MWD, ↓ CRP, ↓ HF events	Estimated difference in KCCQ-CSS 7.8 points (95% CI 4.8–10.9, *p* < 0.001) Estimated difference in the 6 min walk distance 20.3 m (95% CI 8.6–32.1, *p* < 0.001)
STEP-HFpEF DM (2024) [65]	Obese HFpEF + T2DM	Semaglutide	Similar benefit to STEP-HFpEF; smaller weight loss ↓ NT-proBNP	Estimated difference in KCCQ-CSS 7.3 points (95% CI 4.1–10.4, *p* < 0.001) Estimated difference in the 6 min walk distance 14.3 m (95% CI 3.7–24.9, *p* = 0.008) HR for hospitalization or urgent visit for HF 0.40 (95% CI 0.15–0.92)
STEP-HFpEF Pooled Analysis (2024) [66]	Combined STEP trials	Semaglutide	Greater benefit in severe HFpEF ↓ CRP	Mean between-group difference in KCCQ-CSS 7·5 points (95% CI 5.3–9.8) Mean between-group difference in 6 min walk distance 17.1 m (95% CI 9.2–25.0)
Patel et al., (2024) [68]	HFpEF + T2DM + Obesity	GLP-1RA + SGLT2i	Combination therapy better than SGLT2i alone	HR for HF exacerbations 0.62 (95% CI 0.58–0.66, *p* < 0.001) HR for AKI events 0.70 (95% CI 0.65–0.75, *p* < 0.001) HR for new onset of AF/AFL 0.81 (95% CI 0.70–0.95, *p* = 0.007)
SUMMIT (2025) [19]	Obese HFpEF ± T2DM	Tirzepatide	↓ CV death or worsening HF; ↑ 6MWD, ↓ CRP	HR for CV deaths or a worsening heart-failure event 0.62 (95% CI 0.41–0.95, *p* = 0.026)

↑ Increase ↓ Decrease.

## Data Availability

No new data were created or analyzed in this study. Data sharing is therefore not applicable to this article.
